# Phosphorylation of Nicastrin by SGK1 Leads to Its Degradation through Lysosomal and Proteasomal Pathways

**DOI:** 10.1371/journal.pone.0037111

**Published:** 2012-05-10

**Authors:** Jung-Soon Mo, Ji-Hye Yoon, Ji-Ae Hong, Mi-Yeon Kim, Eun-Jung Ann, Ji-Seon Ahn, Su-Man Kim, Hyeong-Jin Baek, Florian Lang, Eui-Ju Choi, Hee-Sae Park

**Affiliations:** 1 School of Biological Sciences and Technology, Hormone Research Center, Chonnam National University, Gwangju, Republic of Korea; 2 Department of Physiology, University of Tübingen, Tübingen, Germany; 3 School of Life Sciences and Biotechnology, Korea University, Seoul, Republic of Korea; University Paris Sud, France

## Abstract

The gamma-secretase complex is involved in the intramembranous proteolysis of a variety of substrates, including the amyloid precursor protein and the Notch receptor. Nicastrin (NCT) is an essential component of the gamma-secretase complex and functions as a receptor for gamma-secretase substrates. In this study, we determined that serum- and glucocorticoid-induced protein kinase 1 (SGK1) markedly reduced the protein stability of NCT. The SGK1 kinase activity was decisive for NCT degradation and endogenous SGK1 inhibited gamma-secretase activity. SGK1 downregulates NCT protein levels via proteasomal and lysosomal pathways. Furthermore, SGK1 directly bound to and phosphorylated NCT on Ser437, thereby promoting protein degradation. Collectively, our findings indicate that SGK1 is a gamma-secretase regulator presumably effective through phosphorylation and degradation of NCT.

## Introduction

The gamma-secretase complex is involved in the overproduction of amyloid-beta peptide (Abeta), a hallmark of Alzheimer's disease (AD) [1], [2], [3]. The principal component of amyloid plaques, Abetais generated from amyloid precursor protein (APP) by beta- and gamma-secretase. Gamma-secretase is a high-molecular-weight multimeric protein complex with aspartyl protease activity that is responsible for the cleavage of several type I transmembrane proteins, including amyloid precursor protein (APP) and the Notch receptor [4], [5]. Gamma-secretase is composed of four transmembrane proteins: Presenilin 1 (PS1), Nicastrin (NCT), Presenilin enhancer 2 (PEN-2), and anterior pharynx-defective-1 (APH-1) [1], [5], [6], [7], [8], [9], [10], [11], [12], [13], [14], [15], [16]. PS1 is generally recognized as the catalytic core protein of the complex [17]. NCT is important for the stability and trafficking of other gamma-secretase components, and is pivotal in the stabilization of PS1 expression and the creation of a substrate docking site in the complex [1], [18], [19], [20], [21]. APH-1, a multi-transmembrane domain protein, is thought to stabilize the gamma-secretase complex (operating in conjunction with NCT); PEN-2 may cause a conformational change in NCT and also be important in the endoproteolysis of PS during the maturation of the complex [1], [22], [23], [24], [25].

The NCT gene is located on chromosome 1q23, a region that is linked to an AD susceptibility locus [26]. NCT performs a critical function in gamma-secretase complex activation and in the Abeta generation associated with AD pathogenesis [1], [5], [27], [28]. NCT is a 709-amino acid single-pass membrane protein, and is the most abundant subunit of the gamma-secretase complex; the protein harbors a number of glycosylation sites within its large extracellular domain (ECD) [11], [29]. NCT is synthesized in fibroblasts and neurons as an endoglycosidase-H-sensitive glycosylated precursor protein (immature NCT). Immature NCT is modified by complex glycosylation to generate the mature NCT in the Golgi [29], [30]. NCT is a member of the amino-peptidases/transferrin receptor superfamily implying that NCT a catalytic or a binding role in APP processing [11]. NCT degradation is accomplished by both lysosomal and proteasomal pathways [31]. According to recent evidence Synoviolin (also referred to as Hrd1), an E3 ubiquitin ligase implicated in endoplasmic reticulum-associated degradation, is involved in the degradation of immature NCT [32]. The half-life and activity of NCT are regulated primarily by its phosphorylation by ERK, JNK, and possibly other kinases [33], [34]. However, little is currently known regarding any other protein kinase(s) that might contribute to the turnover of NCT.

The serum- and glucocorticoid-induced kinase 1 (SGK1), SGK1, is a serine/threonine kinase downstream of the PI3K cascade [35]. SGK1 is a member of the AGC family of protein kinases, including protein kinases A, G, and C, and is related to the major cellular survival factor, protein kinase B (PKB, also called Akt). SGK1 and PKB share 45% to 55% homology within their catalytic domain [36], [37], [38]. In mammalian cells, two more isoforms of SGK1 have been described, referred to as SGK2 and SGK3 [37]. They share 80% homology in their catalytic domains and are evolutionally conserved. The expression of SGK1, but not SGK2 or SGK3, is acutely regulated by glucocorticoids and serum [39]. Similar to several other AGC kinases, SGK1 is activated via stimulation by 3-phosphoinositide-dependent kinase 1/2-mediated phosphorylation and is tightly linked to the phosphatidylinositol 3-kinase pathway (PI3K) dependent cell survival pathway. SGK1 is regulated at both the transcriptional and posttranslational levels by external stimuli including hepatocyte growth factor as well as steroid hormones, particularly aldosterone and growth factors like insulin [36], [37], [38], [40], [41], [42]. SGK and Akt are thought to phosphorylate related substrates, because they share a similar consensus phosphorylation site (RXRXXS/T) [39].

Recently, we disclosed that SGK1 downregulates the protein stability of the Notch1 intracellular domain, which is cleaved proteolytically by gamma-secretase via Fbw7 E3 ubiquitin ligase phosphorylation, thereby suggesting that SGK1 modulates Notch1 activity in a gamma-secretase independent manner [43]. In the present study, we elucidated the role of SGK1 in the regulation of gamma-secretase and APP processing. We demonstrate herein that SGK1, when activated, inhibits the cleavage of APP. SGK1 physically interacts with and phosphorylates NCT, thereby facilitating protein degradation via proteasomal and lysosomal pathways. We also report that dexamethasone, which induces SGK1 expression in various cell types, inhibited gamma-secretase activity and stimulated the phosphorylation and degradation of NCT.

## Results

### SGK1 downregulates gamma-secretase-dependent APP cleavage

**Figure 1:**
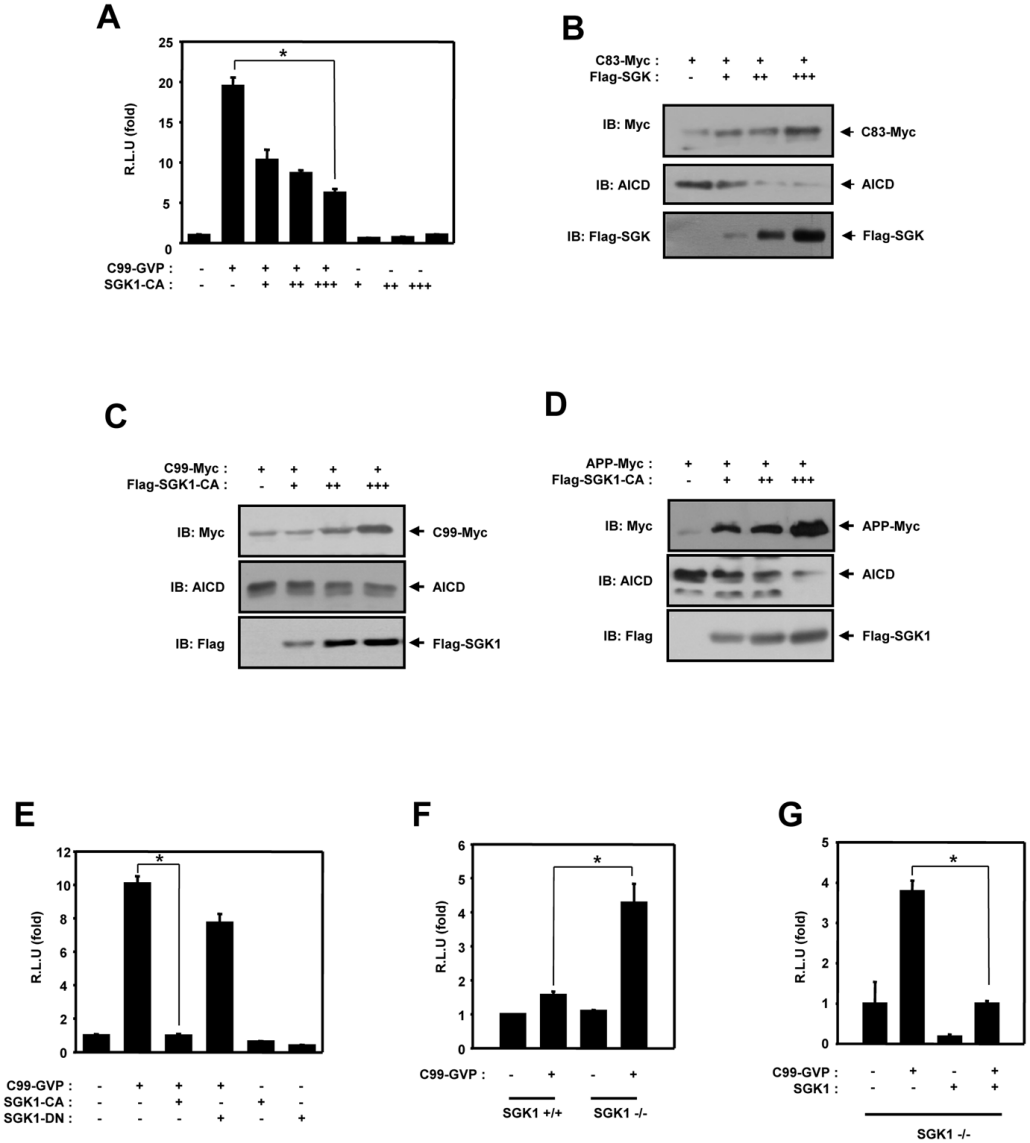
SGK1 downregulates gamma-secretase dependent APP cleavage. (A) (A) HEK293 cells were transfected with expression vectors encoding for 100 ng of GAL4-Luc, 100 ng of beta-galactosidase, along with 200 ng of C99-Gal4/VP16 and 200 ng(+), 400 ng(++) and 600 ng(+++) of SGK1-CA, as indicated. (B) HEK293 cells were transfected for 48 hours with expression vectors encoding for 500 ng of C99-Myc, along with 500 ng (+), 1 µg (++) and 1.5 µg (+++) of Flag-SGK1 CA, as indicated. (C) HEK293 cells were transfected for 48 hours with expression vectors encoding for 500 ng of C99-Myc, along with 500 ng (+), 1 µg (++) and 1.5 µg (+++) of Flag-SGK1 CA, as indicated. (D) HEK293 cells were transfected for 48 hours with expression vectors encoding for 500 ng of APP695, along with 500 ng (+), 1 µg (++) and 1.5 µg (+++) of Flag-SGK-CA. (B, C, D) The cell lysates were immunoblotted with anti-Myc (9E10), anti-APP c-terminal and anti-Flag antibodies (E) HEK293 cells were transfected with expression vectors encoding for 200 ng of C99-Gal4/VP16, 100 ng of GAL4-Luc, 100 ng of beta-galactosidase, 600 ng of SGK1-CA and SGK1-DN. (F) MEF cells from SGK1_+/+_ and SGK1_−/−_ mice were transfected with expression vectors encoding for 200 ng of C99-Gal4/VP16 and 100 ng of beta-galactosidase with GAL4-Luc (G) MEF cells from SGK1_−/−_ mice were transfected with expression vectors encoding for 100 ng of GAL4-Luc, 100 ng of beta-galactosidase and 200 ng of HA-SGK1 with 100 ng of C99-Gal4/VP16. (A, E–G) After 48 hours of transfection, the cells were lysed and the luciferase activity was assayed. Data were normalized to beta-galactosidase activity. The results are expressed as the means ± SEM from three independent experiments. (B–D) These results represent one of three independent experiments. RLU means relative luciferase units. The data were evaluated for significant difference using the Student's *t*-test; ANOVA, *P<0.001. IB, Immunoblot.

Processing of the amyloid precursor protein results in the generation of the amyloidogenic peptide, Abeta, which plays a central role in the pathogenesis of Alzheimer's disease. Cleavage of C99, the APP c-terminal fragment derived from beta-secretase processing of APP, by gamma-secretase generates the Abeta peptide. Furthermore, gamma-secretase cleavage of C99 and C83, the alpha-secretase derived APP C-terminal fragment (APP CTF), releases the APP C-terminal domain (AICD), a 6-kDA peptide also called CTD or AID, which regulates transcription after translocation to the nucleus. The alpha-secretase and beta-secretase initially cleave APP. Cleavage of APP by alpha-secretase generates the C83. C99 derives from its precursor APP through proteolytic events that are mediated by beta-secretase. Thereafter C83 and C99 is a direct substrate of gamma-secretase [33], [44]. Because APP is cleaved by gamma-secretase activity, we first attempted to determine whether or not SGK1 plays a role in the regulation of APP-mediated signaling. We used C99-Gal4/VP16 fusion proteins to measure the gamma-secretase-induced cleavage of APP [45], [46], [47]. HEK293 cells were transfected with C99-Gal4/VP16 and pG5E1BLuc, as well as either SGK1 or an empty vector. As expected, APP-mediated reporter activity increased in these samples (Fig. 1A). SGK1 exerted no influence on the basal levels of Gal4-Luc activity but attenuated the ability of C99-Gal4/VP16 to stimulate reporter activity in a dose-dependent manner (Fig. 1A). To determine whether gamma-secretase-mediated APP cleavage is regulated by SGK1, thereby promoting AICD production, we conducted western blot with HEK293 cells using APP and, the C83 and the C99 form of APP as a direct substrate of gamma-secretase and SGK1. To determine whether SGK1 is involved in regulating gamma-secretase-mediated APP cleavage, Western blotting was conducted with HEK293 cells using the C99 form of APP as a substrate for gamma-secretase and SGK1. As a result, SGK1 reduced the gamma-secretase dependent cleavage of C83 (Fig. 1B), C99 (Fig. 1C) and APP (Fig. 1D) in a dose-dependent fashion. C83, C99 and APP proteins were elevated, whereas AICD was evidently decreased (Fig. 1B–D). These results indicated that SGK1 suppresses the gamma-secretase-dependent cleavage of APP in intact cells.

### Loss of function of SGK1 rescues gamma-secretase dependent cleavage of APP

In an effort to determine whether the kinase activity of SGK1 is required for the downregulation of gamma-secretase-dependent APP cleavage, we used a constitutively active form of SGK1 (SGK1-CA) and a dominant-negative mutant of SGK1 (SGK1-DN) to block the kinase activity of SGK1. In the luciferase reporter gene assay with HEK293 cells, SGK1-CA and SGK1-DN were transfected and gamma-secretase-dependent cleavage evaluated using C99-Gal4/VP16. The gamma-secretase-dependent luciferase reporter activity was inhibited by SGK1-CA, but was not inhibited by the cotransfection of C99-Gal4/VP16 and SGK1-DN (Fig. 1E). In order to assess the role of endogenous SGK1 in gamma-secretase-dependent APP cleavage, we conducted a Gal4-transactivation assay using SGK1 wild-type (SGK1^+/+^) and SGK1-deficient (SGK1^−/−^) MEF cells. The transactivation of C99-Gal4/VP16 in SGK1- deficient MEF cells was four-fold higher than that observed in the SGK1 wild-type cells (Fig. 1F). We also found that the gamma-secretase-dependent transactivation of C99-Gal4/VP16 was suppressed by SGK1 overexpression in SGK1-deficient cells (Fig. 1G). These results demonstrated that the kinase activity of SGK1 is, indeed, crucially important for the regulation of gamma-secretase-dependent APP cleavage.

### SGK1 prevents the physical interaction between NCT and APP

**Figure 2:**
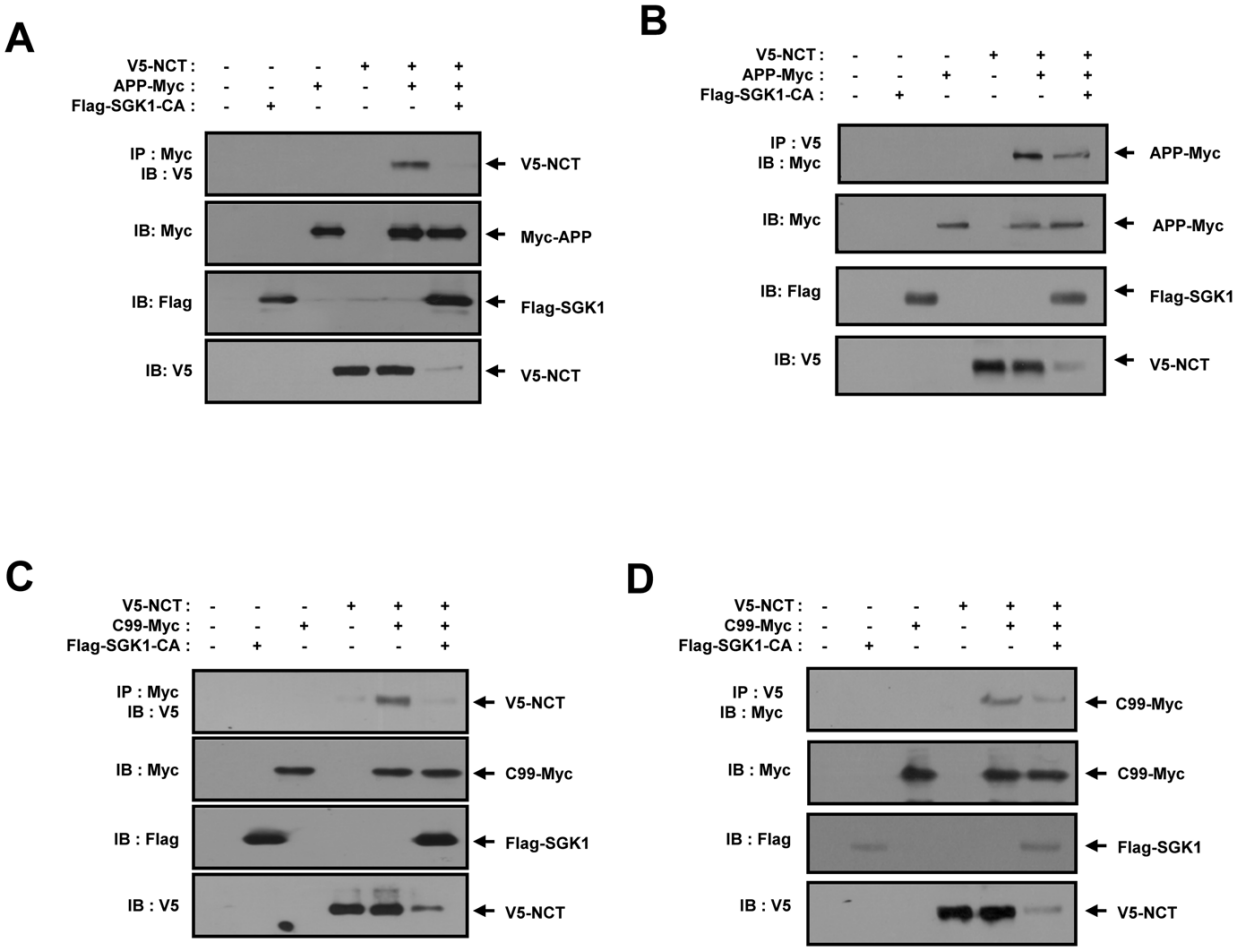
SGK1 prevents the physical interaction between NCT and APP. (A, B) HEK293 cells were transfected for 48 hours with expression vectors encoding for the indicated combinations of 2 µg of V5-NCT, 2 µg of APP-Myc, and 6 µg of Flag-SGK1- CA. (A) Cell lysates were subjected to immunoprecipitation with an anti-Myc antibody (9E10), and the immunoprecipitates were immunoblotted with an anti-V5 antibody. (B) Cell lysates were subjected to immunoprecipitation with an anti-V5 antibody, and the immunoprecipitates were immunoblotted with an anti-Myc antibody (9E10). (C, D) HEK293 cells were transfected for 48 hours with expression vectors encoding for the indicated combinations of 2 µg of V5-NCT, 2 µg of C99-Myc, and 4 µg of Flag-SGK1-CA. (C) Cell lysates were subjected to immunoprecipitation with an anti-Myc antibody (9E10), and the immunoprecipitates were immunoblotted with an anti-V5 antibody. (D) Cell lysates were subjected to immunoprecipitation with an anti-V5 antibody, and the immunoprecipitates were immunoblotted with an anti-Myc antibody (9E10). (A–D) The cell lysates were also subjected to immunoblot analysis with anti-Myc (9E10), anti-Flag, and anti-V5 antibodies, respectively. These results represent one of three independent experiments. IB, Immunoblot. IP, Immunoprecipitation.

To observe the effects of SGK1 on the molecular interactions between NCT and APP, coimmunoprecipitation was conducted in HEK293 cells via the cotransfection of V5-tagged NCT, Myc-tagged APP, and Flag-tagged SGK1-CA. Without coexpression of SGK1, NCT and APP were coimmunoprecipitated. In contrast, when they were cotransfected with SGK1, the band of NCT that interacted with APP disappeared (Fig. 2A). The cell lysates were analyzed via immunoprecipitation with an anti-V5 antibody, and immunoblotting was conducted using the anti-Myc antibody. Conversely, under conditions identical to those described above, immunoblot analysis of the V5 immunoprecipitates with an anti-Myc antibody also showed the interaction between the two proteins (Fig. 2B). Next, HEK293 cells were cotransfected with V5-tagged NCT, a Myc-tagged C99 form of APP, and Flag-tagged SGK, and coimmunoprecipitation was performed. Interestingly, when the cells were cotransfected with SGK1, the band of NCT that interacted with C99 disappeared (Fig. 2C, D). Surprisingly, on the cell lysate immunoblot, the levels of mature and immature forms of NCT protein were downregulated upon cotransfection with SGK1 (Fig. 2A–D). This finding strongly suggests that SGK regulates NCT protein levels.

### SGK1 down-regulates the protein level of NCT through proteasomal and lysosomal degradation

**Figure 3:**
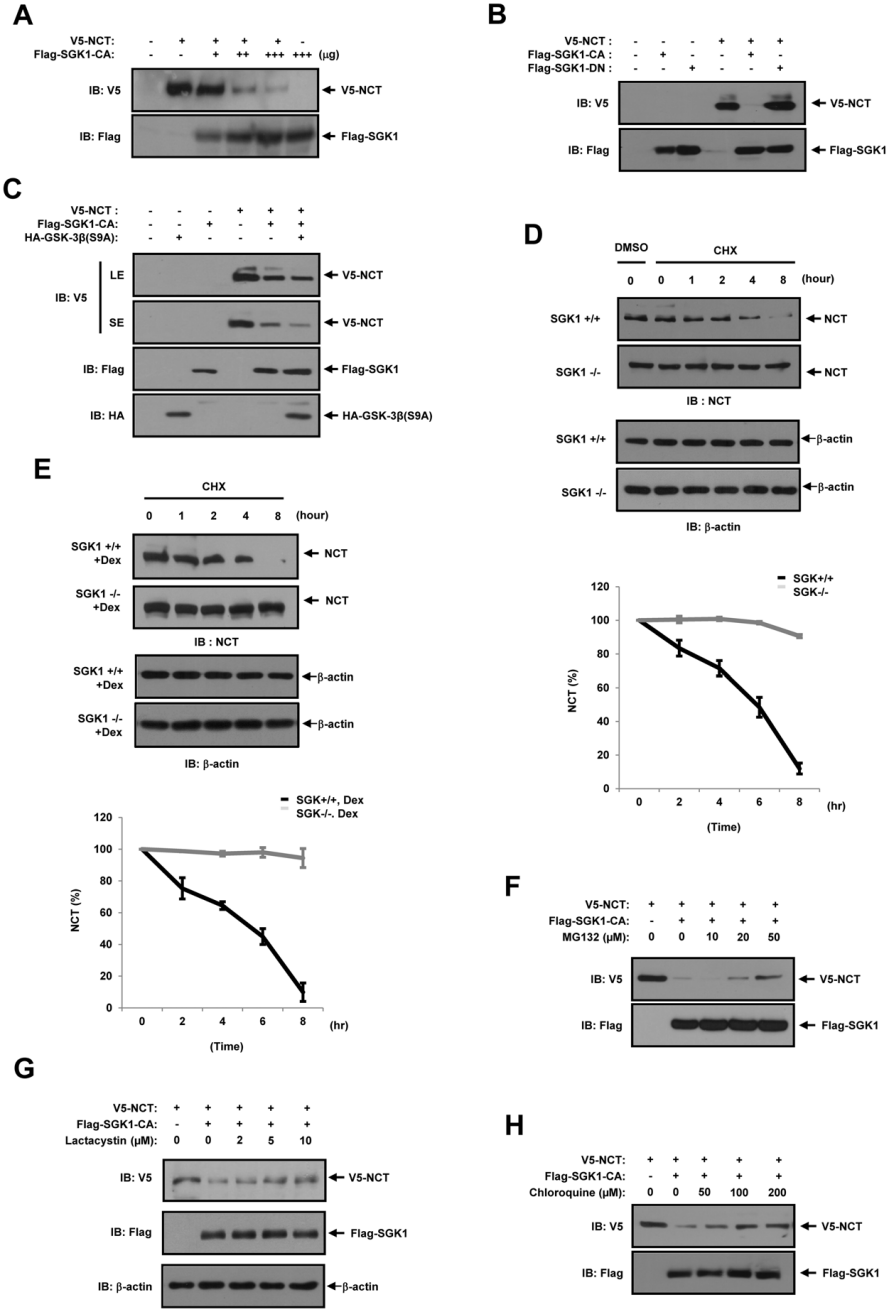
SGK1 downregulates the NCT protein levels via proteasomal and lysosomal degradation. (A) HEK293 cells were transfected for 48 hours with expression vectors encoding for 0.5 µg of V5-NCT and 0.5 µg (+), 1 µg (++) and 1.5 µg (+++) of Flag-SGK1 CA, as indicated. The cell lysates were immunoblotted with anti-Flag and anti-V5 antibodies. (B) HEK293 cells were transfected for 48 hours with expression vectors encoding for 1 µg of V5-NCT, 2 µg of Flag-SGK1-CA, and 2 µg of Flag-SGK1-DN. The cell lysates were immunoblotted with anti-Flag, and anti-V5 antibodies. (C) HEK293 cells were transfected for 48 hours with expression vectors encoding for 1 µg of V5-NCT, 2 µg of Flag-SGK1-CA, and 2 µg of HA-GSK-3beta(S9A). The cell lysates were immunoblotted with anti-HA, anti-Flag, and anti-V5 antibodies. NCT blots are represented as short (SE) or long (LE) exposures. (D) SGK1^+/+^ and SGK1^−/−^ MEF cells were treated with 100 µM DMSO or 100 µM cycloheximide (CHX) for the indicated periods of time, and the cell lysates immunoblotted with anti-Notch1-IC antibody (left). We quantified the intensity of each band using a densitometer and plotted relative intensities (right). (E) SGK1^+/+^ and SGK1^−/−^ MEF cells were treated with Dexamethasone (1 µM) for 12 hours, and cell lysates immunoblotted with anti-NCT antibody. (F) HEK293 cells were transfected for 48 hours with expression vectors encoding for 1 µg of V5-NCT and 3 µg of Flag-SGK1-CA. HEK293 cells were treated with various concentrations (0, 10, 20, 50 µM) of MG132 for 6 h. (G) HEK293 cells were transfected for 48 hours with expression vectors encoding for 1 µg of V5-NCT and 3 µg of Flag-SGK1-CA. HEK293 cells were treated with various concentrations (0, 2, 5, 10 µM) of Lactacystin for 6 h. (H) HEK293 cells were transfected for 48 hours with expression vectors encoding for 1 µg of V5-NCT and 3 µg of Flag-SGK1-CA. HEK293 cells were treated with various concentrations (0, 50, 100, 200 µM) of Chloroquine for 6 h. The cell lysates were immunoblotted with anti-Flag and anti-V5 antibodies. (A–H) These results represent one of three independent experiments. IB, Immunoblot.

We subsequently subjected HEK293 cells in order to determine whether or not SGK1 performs a role in the regulation of NCT protein levels. The cells were cotransfected with V5-tagged NCT and Flag-tagged SGK1-CA. We detected a dose-dependent reduction in NCT protein levels upon cotransfection of SGK1 (Fig. 3A). The cells were cotransfected with V5-tagged NCT, Flag-tagged SGK1-CA, and Flag-tagged SGK1-DN. Our results showed, also, that NCT protein levels were reduced upon cotransfection with SGK1-CA, but not upon cotransfection with SGK1-DN (Fig. 3B). This suggests that the kinase activity of SGK1 is, indeed, essential for the regulation of NCT protein levels. SGK1 has previously been reported to phosphorylate glycogen synthase kinase (GSK)-3beta on Ser9, resulting in an inactivation of GSK-3beta kinase activity. SGK1 is activated in a phosphoinositide 3-kinase dependent manner, activated SGK1 phosphorylates and thus inhibits, GSK-3beta [48], [49]. GSK-3betais required for gamma-secretase activity and has a role in APP processing [50]. Therefore, using GSK-3beta(S9A), we attempted to determine whether SGK1 downregulates NCT protein levels via GSK-3beta. To evaluate the role played by GSK-3beta in the SGK1-mediated downregulation of NCT protein, HEK293 cells were transfected with GSK-3beta (S9A) and SGK1-CA. Our results indicated that the downregulation of NST protein by SGK1 occurred independently of GSK-3beta (Fig. 3C). Thus, the downregulation of NCT protein levels by SGK1 was shown to be dependent on the intact kinase activity of SGK1, but independent of the downstream kinase, GSK-3beta. We showed that SGK1 downregulates NCT protein levels. The half-life of endogenous NCT was determined using cycloheximide, an inhibitor of protein translation. SGK1 wild-type and SGK1-deficient fibroblast cells were exposed to cycloheximide, and the amount of NCT was analyzed using immunoblotting. The steady-state level and the half-life of endogenous NCT were higher in SGK1-deficient fibroblasts than in wild-type cells (Fig. 3D). Glucocorticoid receptor induces SGK1 expression in a variety of cell lines. We hypothesized that glucocorticoids can contribute to the regulation of NCT protein level through the induction of SGK1. Therefore, the half-life of endogenous NCT was determined using dexamethasone and cycloheximide. SGK1 wild-type and SGK1-deficient fibroblast cells were exposed to dexamethasone and cycloheximide, and the amount of NCT was analyzed using immunoblotting. The steady-state level and the half-life of endogenous NCT were higher in SGK1-deficient fibroblasts than in wild-type cells (Fig.3 E). In order, then, to determine whether the degradation of NCT proteins by SGK1 is mediated by the proteasomal or lysosomal pathway, we conducted test treatments with a proteasomal inhibitor and a lysosomal inhibitor. HEK293 cells were transfected with expression vectors encoding for V5-tagged NCT, or Flag-tagged SGK. HEK293 cells were treated with proteasomal inhibitors MG132 or Lactacystin for 6 hours. As shown in Fig. 3F and G, NCT protein levels were reduced in the presence of SGK1, which were restored by MG132 or Lactacystin treatment in a dose-dependent manner. HEK293 cells were transfected with expression vectors encoding for V5-tagged NCT, or Flag-tagged SGK. HEK293 cells were treated for 6 hours with various concentrations (0, 50, 100, 200 µM) of chloroquine, a lysosomal inhibitor. As shown in Fig. 3H, NCT protein levels were rescued by chloroquine in a dose-dependent manner. These results indicate that the stability of the NCT protein was downregulated by SGK1 by a mechanism requiring both the proteasomal and lysosomal pathways.

### The involvement of SGK1 in the dexamethasone-induced downregulation of the NCT protein levels

**Figure 4:**
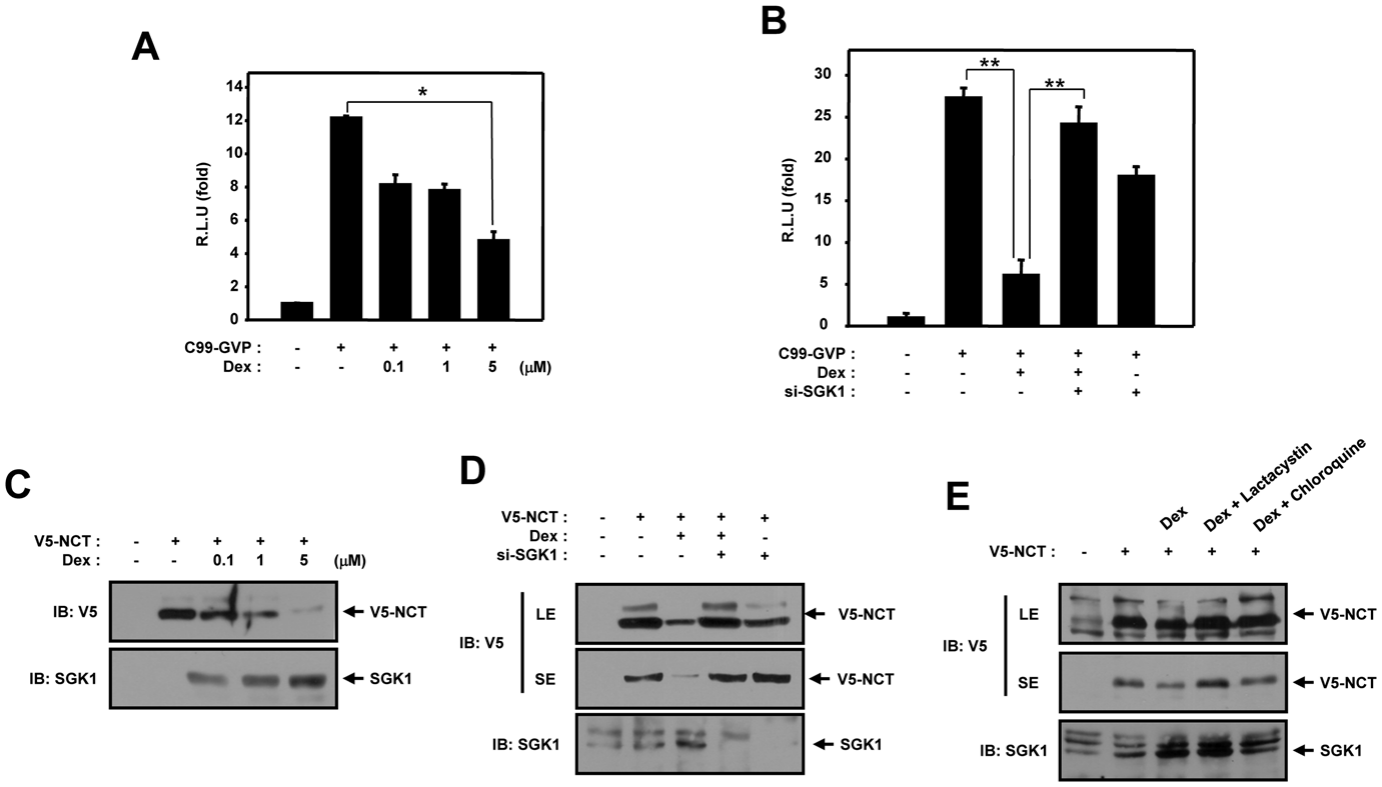
Induced SGK1 by dexamethasone downregulates the NCT protein levels. (A) HEK293 cells were transfected with expression vectors encoding for 200 ng of C99-Gal4/VP16, 100 ng of GAL4-Luc with 100 ng of beta-galactosidase and exposed to 0.1, 1, 5 µM dexamethasone for 24 hours. (B) HEK293 cells were transfected with expression vectors encoding for 200 ng of C99-Gal4/VP16, 100 ng of GAL4-Luc, 100 ng of beta-galactosidase with 400 ng of siSGK1 and exposed to 1 µM dexamethasone for 24 hours. After 48 hours of transfection, the cells were lysed and the luciferase activity was assayed. Data were normalized to beta-galactosidase activity. The results are expressed as the means ± SEM from three independent experiments. RLU means relative luciferase units. The data were evaluated for significant difference using the Student's *t*-test; ANOVA, *P<0.05, ** *P*<0.001. (C) HEK293 cells were transfected for 48 hours with expression vectors encoding for 1 µg of V5-NCT and exposed to 0.1, 1 and 5 µM dexamethasone for 24 hours. (D) HEK293 cells were transfected for 48 hours with expression vectors encoding for 1 µg of V5-NCT and 2 µg of si-SGK1and exposed to 5 µM dexamethasone for 24 hours. The cell lysates were immunoblotted with anti-V5 and anti-SGK1 antibodies. NCT blots are represented as short (SE) or long (LE) exposures. (E) HEK293 cells were transfected for 48 hours with expression vectors encoding for 1 µg of V5-NCT and pretreated with 1 µM dexamethasone for 24 hours. The cells were pretreated with Dexamethasone. After incubation with 10 µMof Lactacystin or 200 µM of chloroquine for 6 hr, the cells were harvested and the cell lysates were immunoblotted with anti-V5 and anti-SGK1 antibodies. NCT blots are represented as short (SE) or long (LE) exposures. (C–E) These results represent one of three independent experiments. RLU means relative luciferase units. IB, Immunoblot.

Glucocorticoid induces SGK1 expression in a variety of cell lines. In an effort to characterize the effect of dexamethasone on the gamma-secretase-dependent cleavage of APP, we conducted a luciferase reporter gene assay, using HEK293 cells. When the cells were treated for 24 hours with 0.1, 1, or 5 µM dexamethasone, the gamma-secretase dependent processing of APP was suppressed, and this effect occurred in a dose-dependent manner (Fig. 4A). In the gamma-secretase-dependent luciferase reporter gene assay with HEK293 cells, APP and si-SGK1 were transfected and the cells were treated for 24 hours with 1 µM dexamethasone. The gamma-secretase-dependent processing was suppressed by dexamethasone, but the gamma-secretase-dependent APP cleavage was rescued by co-transfection with si-SGK1 (Fig. 4B). The effect of this transfection on the gamma-secretase dependent procession of APP was then assessed using Gal4-Luc. We next attempted to ascertain whether dexamethasone was able to facilitate the degradation of NCT in intact cells. HEK293 cells were transfected with V5-tagged NCT and exposed to dexamethasone. The immunoblot data demonstrated that dexamethasone induces the degradation of NCT protein levels in a dose-dependent manner (Fig. 4C). The dexamethasone-induced degradation of NCT was rescued significantly by coexpression with SGK1 siRNA (Fig. 4D). Also the NCT protein level was recovered in the presence of the proteasome inhibitor (MG132) or lysosomal inhibitor (Chloroquine) (Fig. 4E).These results imply that SGK1, which is induced by dexamethasone, negatively regulated gamma-secretase dependent APP cleavage via degradation of NCT protein through the proteasome or lysosomal dependent pathway in intact cells.

### NCT interacts directly with SGK1 in intact cells

**Figure 5:**
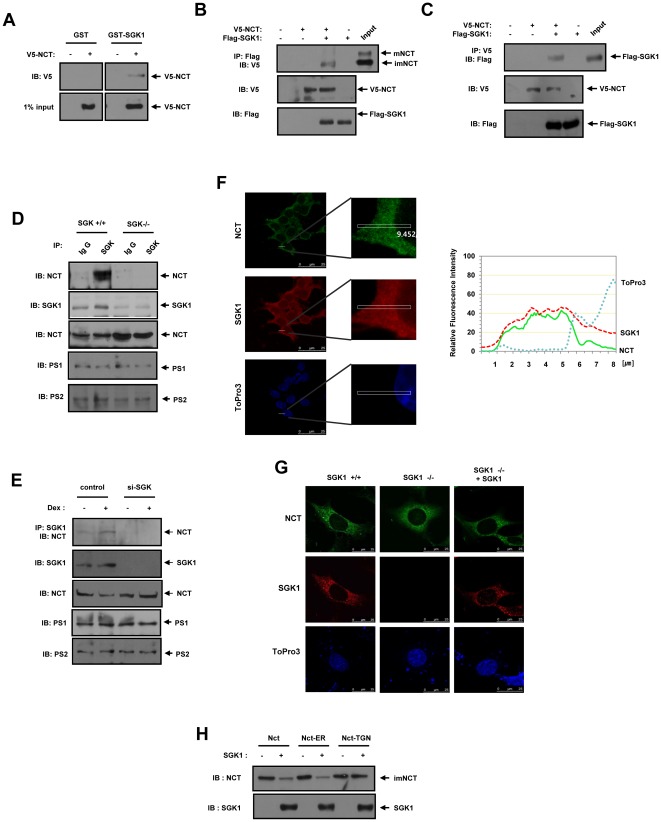
NCT interacts directly with SGK1 in intact cells. (A) Recombinant GST or GST–SGK1 proteins were immobilized onto GSH-agarose. HEK293 cells were transfected with an expression vector encoding for 2 µg of V5-NCT or an empty vector. After 48 hours of transfection, the cell lysates were subjected to GST pull-down experiments with immobilized GST or GST–SGK1. Proteins bound to GST or GST–SGK1 was analyzed via immunoblotting with an anti-V5 antibody. The input represents 1% of the cell lysate prior to *in vitro* binding assay. (B, C) HEK293 cells were transfected with expression vectors encoding for 2 µg of V5-NCT and 2 µg of Flag-SGK1 as indicated. (B) After 48 hours, the cell lysates were subjected to immunoprecipitation (IP) with anti-Flag. The immunoprecipitates were then immunoblotted (IB) with anti-V5. (C) Cell lysates were subjected to immunoprecipitation with an anti-V5 antibody, and the immunoprecipitates were immunoblotted with an anti-Flag antibody. (D) MEF cells from SGK1_+/+_ and SGK1_−/−_ mice were lysed and subjected to immunoprecipitation with immunoglobulin G (IgG) and anti-SGK1 antibodies as indicated. Immunoprecipitates were immunoblotted with an anti-NCT antibody. (E) Rat fibroblast Rat2 cells expressing either control siRNA or rSGK1 siRNA were left untreated or treated with 1 µM dexamethasone for 24 hours. Rat fibroblast Rat2 cells expressing either control siRNA or rSGK1 siRNA were lysed and subjected to immunoprecipitation with anti-SGK antibody. Immunoprecipitates were immunoblotted with an anti-NCT antibody. (F, G) MEF from SGK1^+/+^, SGK1^−/−^ mice or HEK293 cells or were stained with Alexa 488 (green) and Alexa 546 (red) and examined by confocal microscopy. The DNA dye ToPro3 was used to visualize nuclei of all cells. For each experiment, at least 200 cells were examined, and the figures shown here represent the typical staining pattern for a majority of cells and quantify the fold enrichment at the indicated region (white box). (H) HEK293 cells were transfected for 48 hours with expression vectors encoding for 1 µg of NCT, NCT-ER, NCT-TGN and 2 µg of Flag-SGK1. The cell lysates were immunoblotted with anti-Flag, and anti-NCT antibodies. (A–E, H) The cell lysates were also subjected to immunoblotting analysis with the indicated antibodies. These results represent one of three independent experiments. IB, Immunoblot. IP, Immunoprecipitation.

Considering that our results implicate NCT as a target of SGK1, we then attempted to determine whether these two proteins interact directly in intact cells. In the *in vitro* binding studies, purified GST and GST–SGK1 proteins were immobilized on GSH agarose. Cell lysates expressing V5-tagged NCT were incubated either with immobilized GST or with GST–SGK1 on GSH-agarose. The interaction between GST–SGK1 and NCT was then detected on bead complexes (Fig. 5A). HEK293 cells were cotransfected with vectors encoding for V5-tagged wild-type NCT and Flag-tagged SGK1 and subsequently subjected to coimmunoprecipitation analysis (Fig. 5B, C). Immunoblot analysis of the Flag immunoprecipitates from the transfected cells with an anti-V5 antibody revealed that V5-NCT was physically associated with Flag-SGK1 in the cells (Fig. 5B). By contrast, immunoblot analysis using anti-Flag antibody of the V5-immunoprecipitates also showed interaction between the two proteins (Fig. 5C). We also attempted to ascertain whether or not endogenous NCT and SGK1 could interact in intact cells. Using MEF cells acquired from SGK1^+/+^ and SGK1^−/−^ mice, an immunoblot analysis of the SGK1 immunoprecipitates with an anti-NCT antibody showed direct association between endogenous NCT and SGK1 in SGK1^+/+^ cells (Fig. 5D). Using Rat fibroblast Rat2 cells expressing control siRNA or rSGK1 siRNA, immunoblot analysis of the SGK1 immunoprecipitates with an anti-NCT antibody revealed that endogenous SGK1 and NCT associated physically in dexamethasone treated Rat fibroblast Rat2 cells (Fig. 5E). SGK1 is distributed throughout both the cytoplasm and nucleus [51]. NCT has been reported to be localized in the lysosomal membrane, plasma membrane and trans-Golgi network in the cytoplasm [52], [53], [54], [55]. We compared endogenous SGK1 and NCT staining and found that they colocalized in the cytoplasm, but not in the nucleus (Fig. 5F). We also examined that endogenous SGK1 and NCT localization using MEF cells from SGK1^+/+^ and SGK1^−/−^ mice. Endogenous SGK1 and NCT colocalized in the cytoplasm in SGK1^+/+^ cells, but not in SGK1^−/−^ cells (Fig. 5G). Next, to determine whether the NCT mutant where localize the different subcellular compartment could be promoted degradation by SGK1, we expressed NCT variants harboring either an endoplasmic reticulum (ER) retention signal (NCT-ER) or a trans-Golgi network (TGN) targeting motif (NCT-TGN) [56] (Fig. 5H). Immunoblot analysis revealed that NCT or NCT-ER protein levels were reduced in SGK1 transfected cells. However, SGK1 fail to undergo degradation of NCT-TGN, suggesting that the NCT-TGN mutant does not colocalized with SGK1 in the TGN compartment. Previous reports have shown that subcellular localization of SGK1 is predominantly to the ER in resting cells [57], [58], [59]. Furthermore, previous report has shown that the assembly of gamma-secretase complex occurs in the early compartments of the secretory pathway [56]. These results suggest that physical interaction occur between the two endogenous proteins, NCT and SGK1 in ER compartment in intact cells.

### SGK1 phosphorylates NCT on Ser437, which facilitates degradation of Notch1-IC

**Figure 6:**
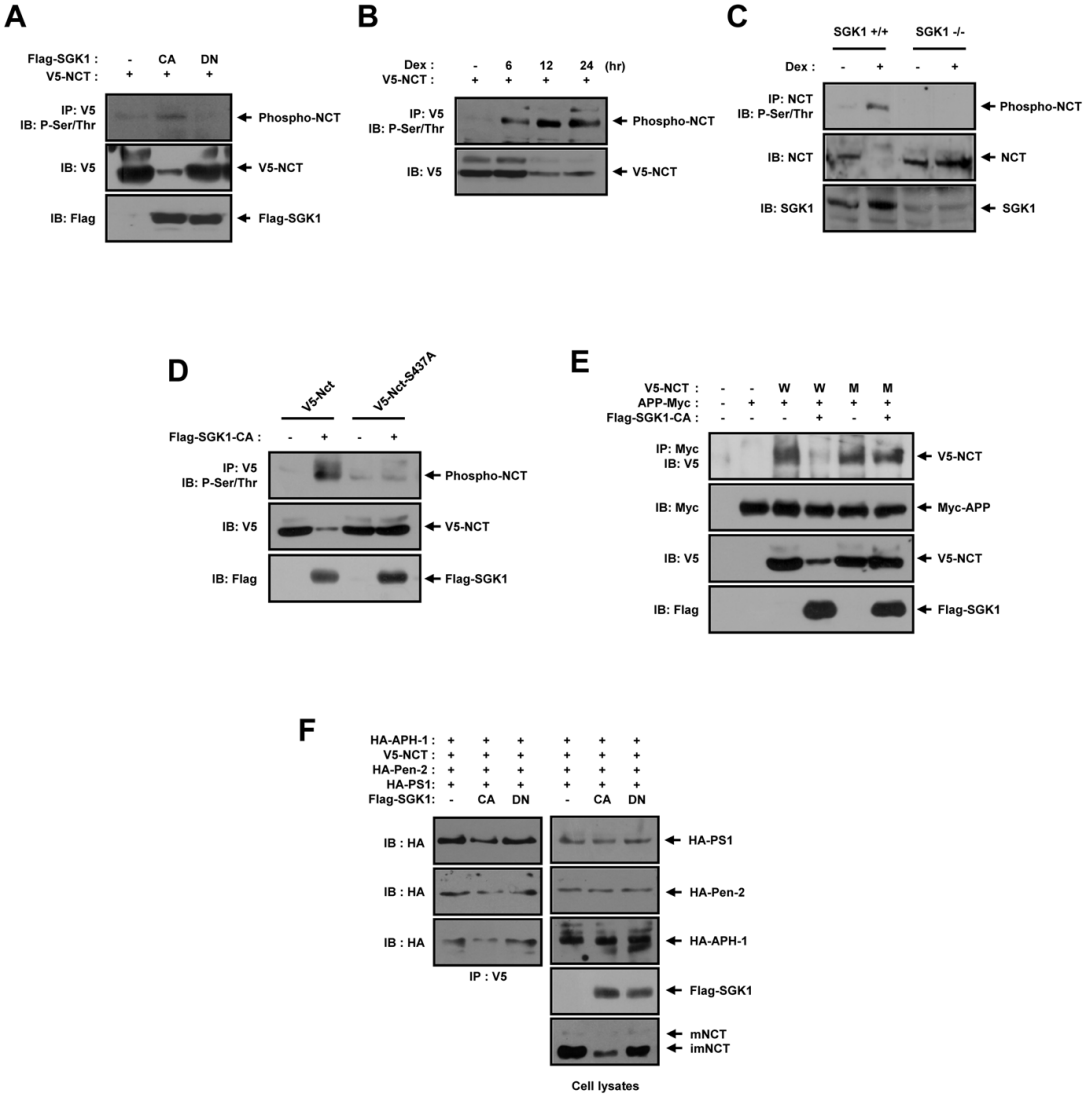
SGK1 phosphorylates NCT on Ser437, which facilitates degradation of NCT. (A) HEK293 cells were transfected with expression vectors encoding for 2 µg of V5-NCT, 4 µg of Flag-SGK1-CA, or Flag-SGK1-DN. After 48 hours of transfection, the cell lysates were subjected to immunoprecipitation with anti-V5 antibody. The immunoprecipitates were immunoblotted with anti-phospho Ser/Thr antibody. (B) HEK293 cells were transfected with expression vectors encoding for 2 µg of V5- NCT, then treated with 1 µM dexamethasone for 24 hours. After 48 hours of transfection the cell lysates were subjected to immunoprecipitation with anti-V5 antibody. The immunoprecipitates were immunoblotted with anti-phospho Ser/Thr antibody. (C) MEF cells from SGK1_+/+_ and SGK1_−/−_ mice were treated with 1 µM dexamethasone for 24 hours. The cell lysates were subjected to immunoprecipitation with an anti-NCT antibody, and the immunoprecipitates were immunoblotted with anti-phospho Ser/Thr antibody. (D) HEK293 cells were transfected with expression vectors encoding for 2 µg of V5-NCT (WT, S437A), 6 µg of Flag-SGK1-CA. After 48 hours of transfection, the cell lysates were subjected to immunoprecipitation with anti-V5 antibody. The immunoprecipitates were immunoblotted with anti-phospho Ser/Thr antibody. (E) HEK293 cells were transfected for 48 hours with expression vectors encoding for the indicated combinations of 2 µg of V5-NCT, 2 µg of V5-NCT (S437A), 2 µg of APP-Myc, and 4 µg of Flag-SGK1-CA. Cell lysates were subjected to immunoprecipitation with an anti-Myc antibody (9E10), and the immunoprecipitates were immunoblotted with an anti-V5 antibody. (F) HEK293 cells were transfected for 48 hours with expression vectors encoding for the indicated combinations of 2 µg of V5-NCT, 2 µg of Flag-SGK1 CA, 2 µg of Flag-SGK1 DN, 2 µg of HA-PS1, 2 µg of HA-Pen2, and 2 µg of HA-APH-1. Cell lysates were subjected to immunoprecipitation with an anti-V5 antibody, and the immunoprecipitates were immunoblotted with an anti-HA antibody. (A–F) The cell lysates were also subjected to immunoblotting analysis with the indicated antibodies. These results represent one of three independent experiments. IB, Immunoblot. IP, Immunoprecipitation.

Next, we conducted Western blot analysis using phosphor Ser/Thr antibody on HEK293 cells to determine whether or not SGK1 plays a role in the phosphorylation of NCT. The cells were cotransfected with V5-tagged NCT, Flag-tagged SGK-CA, and Flag-tagged SGK-DN. The results demonstrated that NCT was phosphorylated upon the cotransfection of Flag-tagged SGK-CA, but was not phosphorylated upon cotransfection of SGK1-DN (Fig. 6A). HEK293 cells were transfected with V5-tagged NCT and treated with 1 µM dexamethasone for 6, 12, and 24 hours. The results showed that the NCT phosphorylation resulting from dexamethasone treatment induced endogenous SGK1 for 24 hours (Fig. 6B). We also conducted Western blot analysis with phospho-Ser/Thr antibody on MEF cells from SGK1^+/+^ and SGK1^−/−^ mice. As a results, endogenous NCT phosphorylation by dexamethasone induced endogenous SGK1 (Fig. 6C). In light of these results, we surmised that the possible phosphorylation sites on NCT were located within a region that harbored the conserved serine residue, Ser437. Via site-directed mutagenesis, we detected the replacement of Ser437 of NCT with alanine. HEK293 cells were cotransfected with vectors encoding for V5-tagged wild-type NCT, V5-tagged NCT S437A point mutant, and Flag-tagged SGK1, and the cells were then subjected to immunoblot analysis (Fig. 6D). Immunoblot analysis from the transfected cells with a phospho-Ser/Thr antibody revealed that only the V5-tagged wild-type NCT was phosphorylated by Flag-SGK1 in the cells. Considering our results implicating NCT Ser437 as the possible phosphorylation site for SGK1, we subsequently attempted to ascertain whether NCT (S437A) is resistant to SGK1-induced degradation, which would imply that the SGK1-induced phosphorylation of NCT is crucial for the degradation of the NCT protein. Immunoblot analysis revealed that NCT Ser437 is resistant to SGK1-induced degradation (Fig. 6D). We then characterized, via coimmunoprecipitation, the involvement of phosphorylation in the physical association between APP and NCT. HEK293 cells were cotransfected with vectors coding for Myc-tagged APP, Flag-tagged SGK1, V5-tagged NCT, and V5-tagged NCT (S437A) and were then subjected to coimmunoprecipitation analysis. Immunoblot analysis of the Myc immunoprecipitates from the cells transfected showed that SGK1 does not prevent the physical association between APP and NCT (S437A) in the cells (Fig. 6E). Next, we examined whether SGK1 kinase activity could influence gamma-secretase complex formation (Fig. 6F), we transfected PS1, Pen-2, NCT and APH1 with SGK1 mutant. Coimmunoprecipitation analysis indicated that gamma-secretase complex formation was effectively suppressed by SGK1-CA, but no SGK1-DN. These results indicate that the phosphorylation of NCT by SGK1 is critically important for its ability to bind to APP due to the degradation of NCT protein.

## Discussion

In this study, we provide the first evidence for a role of SGK1 as endogenous negative regulator of gamma-secretase activity. We further reveal that the kinase activity of SGK1 is essential for SGK1 dependent regulation of gamma-secretase. We demonstrate that SGK1 physically interacts with and phosphorylates NCT and promotes NCT protein degradation via proteasomal and lysosomal pathways. These results provide unequivocal evidence for the involvement of SGK1 in the signaling downregulation of gamma-secretase activity.

NCT is a protein that is part of the gamma-secretase protein complex, along with Presenilin, APH-1, and PEN2 [1]. NCT itself is not catalytically active, but functions as a docking site for gamma-secretase substrates [1], [18]. This study attempted to investigate in greater detail the mechanisms underlying the SGK1-induced modulation of gamma-secretase-dependent APP cleavage. Several recent studies have reported that post-translational NCT modifications, such as glycosylation, palmitoylation, and phosphorylation, are critically important for the regulation of NCT protein stability [29], [33], [34], [60]. NCT is a highly unstable protein, and is degraded via both the proteasomal and lysosomal pathways [31].

Our results revealed that the inhibitory mechanism functioned via the suppression of the interaction of NCT and APP by the downregulation of NCT protein stability; it also appears to be dependent on the kinase activity of SGK1, and independent of GSK-3beta. We determined that SGK1 stimulated the proteasomal degradation of ectopically-expressed NCT, and the reduction of endogenous NCT by SGK1 was also identified as a proteasome-dependent and lysosome-dependent regulatory activity; this indicates that SGK1 modulates gamma-secretase activity by regulating the protein stability of its components. Moreover, when SGK1 activity was inhibited, gamma-secretase activity increased dramatically in cultured cells, including the wild-type or SGK^−/−^ MEF cells. SGK1 activity was disrupted by either blockage of SGK1 expression by small RNA interference or by expression of the dominant-negative mutant SGK1. Under both conditions, the gamma-secretase activity was markedly enhanced. On the other hand, when SGK1 was activated by dexamethasone treatment, the gamma-secretase activity in those cells was reduced substantially.

**Figure 7:**
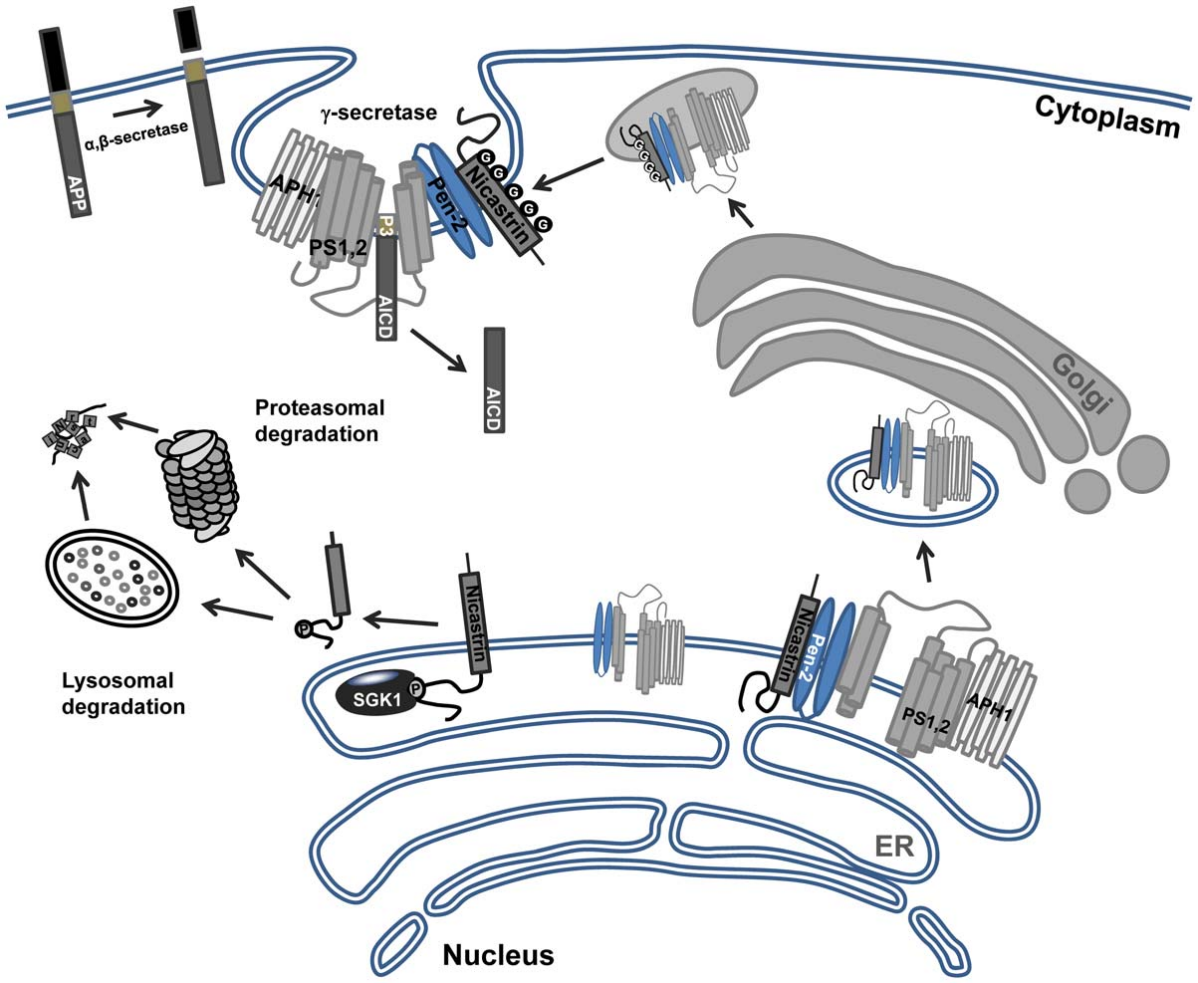
Proposed model for the role of SGK1 in the regulation of NCT protein level. Proteolytic processing of amyloid precursor protein (APP) by the two proteases beta and gamma-secretase controls the generation of the amyloid peptide (Abeta) and the APP intracellular domain. The gamma-secretase complex consists of four essential proteins: presenilin (PS1 or PS2), PEN-2, APH-1 and the nicastrin (NCT). The NCT glycosylation has central role in gamma-secretase assembly and substrate binding. In the glucocorticoid stimulation of SGK expression and activation via glucocorticoid receptor (GR), the immature form of NCT is directly bind and phosphorylated by SGK1, which initiated proteasomal and lysosomal mediated degradation of NCT. Therefore, SGK1 plays a unique and pivotal role in reducing gamma-secretase activity through phosphorylation dependent regulation of NCT protein degradation.

The phosphorylation of NCT by ERK1/2, JNK, and possibly by certain other kinases regulates its gamma-secretase activity in either a positive or negative direction [33], [34]. However, little is currently known regarding any other protein kinase(s) that may participate in NCT turnover. SGK1 preferentially phosphorylates serine and threonine residues that lie within an Arg-Xaa-Arg-Xaa-Xaa-(Ser/Thr) motif [39]. NCT harbors a single phosphorylation consensus sequence for SGK1. SGK1 not only physically interacts with NCT, but also phosphorylates NCT both *in vitro* and in intact cells. Indeed, the results of site-specific mutagenesis demonstrated that SGK1 mediates the phosphorylation of NCT on Ser437, and that this phosphorylation is required for the SGK1-mediated inhibition of NCT. The negative regulation of NCT by SGK1 is further corroborated by our observation that endogenous SGK1, when activated, interacts directly with endogenous NCT in intact cells. Furthermore, we demonstrated that SGK1-mediated NCT phosphorylation on Ser437 results in an increase in the degradation of the NCT protein. Moreover, we determined that SGK1 negatively regulates gamma-secretase activity. Therefore, the interaction between NCT and SGK1 may be one mechanism underlying SGK1-mediated NCT phosphorylation and the proteasomal and lysosomal degradation of NCT (Fig. 7).

SGK1 is known to mediate the intracellular signaling pathway for ion channel conductance, cell volume, and cell survival [36], [37], [38]. Our previous study showed that SGK1 might regulate the stability of the Notch1-IC protein through Fbw7 E3 ligase [43]. In this study, we found that SGK1 directly form a complex with NCT in the ER and accelerating the degradation of NCT by means of phosphorylation, thereby act as an ectodomain kinase of NCT. Previous reports have shown that SGK1 isoforms localize to the luminal side of ER and degraded by the ERAD system in ER, and have the highest level of expression, and their abundance in the ER is regulated by conditions that activate the unfolded protein response (UPR) [57], [58], [59], suggesting that SGK1 can meet their substrates in the ER. NCT translocation occurs through from ER to Golgi and ectodomain of NCT located in the luminal side of ER [52], [53], [54], [55]. Therefore, SGK1 could bind and phosphorylate immature form of NCT ectodomain in the ER. These events fail to catalyze proteolytic conversion of APP and Notch1 into AICD and Notch1-IC, respectively. Therefore, we could suggest the dual regulation of the proteasomal degradation of Notch1-IC by SGK1 via suppression of gamma-secretase and activation of Fbw7 E3 ligase. However, some part of APP and Notch1 could convert into AICD and Notch1-IC via escape from SGK1. We also found that AICD performs the function of a negative regulator in Notch1 signaling by the promotion of Notch1-IC and RBP-Jk protein degradation [61]. The results of this study suggest that SGK1 plays a unique and pivotal role in reducing gamma-secretase activity. Because the activation of SGK1 attenuates gamma-secretase activity, it is expected to reduce Notch1-IC, APP intracellular domain (AICD) and Abeta generation. Therefore, SGK1 may perform dual roles–activating cell survival signals and suppressing Notch1-IC, AICD and Abeta generation.

Hence, the activation of SGK1 might lead to the attenuation of AICD and Abeta-induced neuronal cell death along with other types of brain damage caused by amyloid plaques, such as glial inflammation. Further studies to determine in more detail the relationship between SGK1 and the generation of AICD and Abeta are clearly warranted, and could greatly improve our current understanding of the pathogenesis of AD.

## Materials and Methods

### Cell Culture and Transfection

Human embryonic kidney (HEK) 293 cells (ATCC No. CRL-1573) were grown in Dulbecco's Modified Eagle's Medium (DMEM) supplemented with 10% bovine calf serum (BCS), penicillin (100 U/ml), and streptomycin (100 µg/ml) [43]. Along with the HEK293 cells, Rat fibroblast Rat2 cells expressing either control siRNA or rSGK1 siRNA and Mouse embryonic fibroblasts (MEFs) cells from wild-type or SGK1^−/−^ mice [62] were cultured in Dulbecco's Modified Eagle's Medium (DMEM) supplemented with 10% fetal bovine serum, penicillin (100 U/ml), and streptomycin (100 µg/ml). For transfection with plasmid DNA, the cells were plated at a density of 2×10^6^ cells/100-mm dish, grown overnight, then transfected with the appropriate expression vectors in the presence of the indicated combinations of plasmid DNAs, via the calcium phosphate and liposome method [63].

### Luciferase reporter assay to determine the gamma-secretase activity

The luciferase reporter plasmids were under the control of the Gal4-luciferase reporter plasmid, in either the absence or presence of combinations of expression vectors along with β-galactosidase, in 12-well plates. After 48 hours of transfection, the cells were lysed with chemiluminescent lysis buffer, and were analyzed using a Luminometer (Berthold) for the luciferase assays. The luciferase reporter activity in each sample was normalized in accordance with the beta-galactosidase activity, which had been pre-measured in the samples. For each well of the 12-well pates, C99-GVP, pFR-Luc, beta-galactosidase and absence or presence of combinations of expression vectors were mixed with LipifectAMINE according to the recommendation of the manufacturer (Invitirogen) [45], [46], [47]. After 48 hours of transfection, the cells were lysed with chemiluminescent lysis buffer (18.3% of 1 M K_2_HPO_4_, 1.7% of 1 M KH_2_PO_4_, 1 mM phenylmethyl sulfonyl fluoride (PMSF), and 1 mM dithiothreitol (DTT)). Cell lysates assayed for luciferase activity using a luciferase assay kit (Promega) and were analyzed using a Luminometer (Berthold) for the luciferase assays. The luciferase reporter activity in each sample was normalized in accordance with the beta-galactosidase activity, which had been pre-measured in the samples.

### Western blot analysis

After 48 h of transfection, the cultured HEK293 cells were harvested and lysed for 30 min in RIPA buffer (50 mM Tris–HCl (pH 7.5), 150 mM NaCl, 1% Nonidet P-40, 0.5% Sodium deoxycholate, 0.1% SDS, 1 mM PMSF, 1 mM DTT, and 2 µg/ml leupeptin and aprotinin). The cell lysates were subjected to 20 min of centrifugation at 12,000× g at 4°C. The resultant soluble fraction was then boiled in Laemmli buffer and subjected to SDS–PAGE. After gel electrophoresis, the separated proteins were transferred via electroblotting onto polyvinylidene difluoride (PVDF) membranes (Millipore). The membranes were then blocked with Tris-buffered saline solution (pH 7.4) containing 0.1% Tween 20 and 5% nonfat milk. The blotted proteins were probed with anti-Myc antibody (9E10), anti-HA (12CA5) antibody, or anti-Flag M2 antibody (Sigma Chemical Co.), followed by incubation with anti-mouse horseradish peroxidase-conjugated secondary antibodies (Amersham Biosciences, Inc.). The blots were then developed with an enhanced chemiluminescence (ECL) system (Pierce).

### Coimmunoprecipitation Assays

The cells were lysed in 1 ml of RIPA buffer for 30 minutes at 4°C. After 20 minutes of centrifugation at 12,000× *g*, the supernatants were subjected to immunoprecipitation with the appropriate antibodies coupled to protein A-agarose beads. The resultant immunoprecipitates were then washed three times in phosphate-buffered saline (PBS, pH 7.4). Laemmli sample buffer was added to the immunoprecipitated pellets and the pellets were heated for 5 minutes at 95°C, and then subjected to SDS-PAGE analysis. Western blotting was conducted with the indicated antibodies.

### In vitro binding assay

The recombinant GST-SGK1 protein was expressed in *Escherichia coli* strain BL21, using the pGEX system as indicated. The GST- SGK1 protein was then purified with glutathione-agarose beads (Sigma), in accordance with the manufacturer's instructions. Equal quantities of GST or GST- SGK1 fusion proteins were incubated with the lysates of HEK293 cells, which had been transfected for 3 hours with combinations of expression vectors at 4°C, with rotation. After incubation, the beads were washed three times in ice-cold PBS, and boiled in 20 µl of Laemmli sample buffer. The precipitates were then separated via SDS-PAGE, and the pull-down proteins were detected via immunoblotting with specific antibodies.

### Immunofluorescence staining

Assays were conducted as with HEK293 cells plated at 1×10^5^ cells per well onto cover slips (Fisher). The cultured cells were fixed with 4% paraformaldehyde in phosphate-buffered saline (PBS), and then permeabilized with 0.1% Triton-X 100 in PBS. Cells were blocked in 1% BSA in PBS. Anti-SGK1 antibody (Cell signaling) and anti-NCT antibody (Calbiochem) were employed as the primary antibodies at a dilution of 1∶100, washed twice in PBS. Alexa-488 (Invitrogen) conjugated anti-mouse or Alexa-546 conjugated anti-rabbit secondary antibody (1∶100) was added, and then the DNA dye ToPro3 was used for nuclear localization. The stained cells were evaluated for localization via confocal microscopy (LeicaTCS SPE). Each image is a single Z section at the same cellular level. The final images were obtained and analyzed using confocal microscopy with LAS AF software (Leica). Scale bars represent 25 µm as indicated.

### Site-directed mutagenesis

Site-directed mutagenesis of NCT cDNA was conducted using a Quick Change kit (Stratagene), and the mutagenic primers were S437A (5′-CTTCGAGCTCGAAACATCgGCTGGCGTTGTTCTG-3′) (mismatches with the NCT cDNA template was indicated by lowercase letters). The mutations were verified via automatic DNA sequencing.
